# Developmental Exposure to PCBs, MeHg, or Both: Long-Term Effects on Auditory Function

**DOI:** 10.1289/ehp.0800428

**Published:** 2009-04-03

**Authors:** Brian E. Powers, Emily Poon, Helen J.K. Sable, Susan L. Schantz

**Affiliations:** 1 Neuroscience Program, University of Illinois at Urbana-Champaign, Urbana, Illinois, USA; 2 Psychology Department, University of Memphis, Memphis, Tennessee, USA

**Keywords:** ABR, auditory, development, DPOAE, MeHg, PCB

## Abstract

**Background:**

Developmental exposure to polychlorinated biphenyls (PCBs) or methylmercury (MeHg) can result in a variety of neurotoxic effects, including long-term auditory deficits. However, little is known about the effects of combined exposure to PCBs and MeHg on auditory function.

**Objective:**

We developmentally exposed rats to PCBs and/or MeHg and assessed auditory function in adulthood to determine the effects of exposure to these contaminants individually and in combination.

**Methods:**

We exposed female Long-Evans rats to 1 or 3 mg/kg PCB in corn oil, 1.5 or 4.5 ppm MeHg in drinking water, or combined exposure to 1 mg/kg PCB + 1.5 ppm MeHg or 3 mg/kg PCB + 4.5 ppm MeHg. Controls received corn oil vehicle and unadulterated water. Dosing began 28 days before breeding and continued until weaning at postnatal day (PND) 21. Auditory function of the offspring was assessed at approximately PND 200 by measuring distortion product otoacoustic emissions (DPOAEs) and auditory brainstem responses (ABRs).

**Results:**

Groups exposed to PCBs alone had attenuated DPOAE amplitudes, elevated DPOAE thresholds, and elevated ABR thresholds compared with controls. Groups exposed to MeHg alone did not differ from controls. Unexpectedly, the effects of PCB exposure appeared to be attenuated by coexposure to MeHg.

**Conclusion:**

Developmental exposure to PCBs can result in permanent hearing deficits, and the changes in DPOAE amplitudes and thresholds suggest a cochlear site of action. Coexposure to MeHg appeared to attenuate the PCB-related deficits, but the mechanism for this unexpected interaction remains to be determined.

Polychlorinated biphenyls (PCBs) and methyl-mercury (MeHg) have long been recognized as neurotoxicants that present a potential threat to public health. Humans are exposed primarily via consumption of contaminated fish and seafood. The fetus and neonate are particularly vulnerable to these contaminants because of exposure during critical periods of nervous system development. PCBs and MeHg are readily transferred to the fetus during gestation, and PCBs are also transferred to the neonate through lactation (Rogan et al. 1986a, 1986b). Studies of human populations have identified various adverse outcomes after developmental exposure to PCBs and MeHg, including cognitive, motor, and sensory deficits (Wigle et al. 2008). Numerous studies using animal models have further detailed the nervous system effects of early exposure (Ulbrich and Stahlmann 2004; Watanabe and Satoh 1996).

Studies of auditory function in PCB- or MeHg-exposed humans are limited, but they do suggest that low-level prenatal exposure may impair hearing. Abnormalities of auditory-evoked potentials have been observed in children with early exposure to PCBs, indicating that exposure alters auditory processing in the central nervous system (Chen and Hsu 1994; Vreugdenhil et al. 2004). A study of a PCB-exposed population in the Faroe Islands measured hearing thresholds using traditional audiometric methods at 7 years and found increased thresholds at 250 Hz and 12,000 Hz that were associated with higher prenatal PCB exposure, but only in the left ear (Grandjean et al. 2001). Longnecker et al. (2004) observed a slight increase in hearing threshold at 2,000 Hz in the left ear and 4,000 Hz in the right ear but concluded there was no clinically significant risk of hearing loss associated with pre-natal PCB exposure. Similar auditory deficits have been associated with early MeHg exposure (Murata et al. 1999, 2004), but they observed deficits only in one ear and/or only at specific frequencies, perhaps decreasing confidence in the findings.

Laboratory animal models have provided stronger evidence for PCB- and MeHg-induced auditory deficits. Developmental exposure to the commercial PCB mixture Aroclor 1254 has been demonstrated to result in permanent low-frequency hearing impairment (Goldey et al. 1995; Herr et al. 1996). Further studies indicated that PCB-induced reductions of thyroid hormone concentrations were at least partially responsible for the hearing loss (Goldey and Crofton 1998). The cochlea has been implicated as the likely site of action for the PCB-induced hearing loss. Rats developmentally exposed to Aroclor 1254 exhibited a loss of outer hair cells on the organ of Corti (Crofton et al. 2000). Another study measured distortion product otoacoustic emissions (DPOAEs), which assess the functional integrity of the outer hair cells of the cochlea, and found impairments in rats perinatally exposed to Aroclor 1254 (Lasky et al. 2002). Although these hearing impairments in Aroclor 1254–exposed rats were limited to low frequencies, our previous work has demonstrated hearing impairments across a wide range of frequencies in rats exposed perinatally to an environmental mixture of PCBs (Powers et al. 2006). Exposed rats had attenuated DPOAEs, as well as elevated DPOAE and auditory brainstem response (ABR) thresholds. More recently, Kenet et al. (2007) reported that exposure to PCB-95, a specific *ortho*-substituted PCB congener, disrupted the development of the auditory cortex in rats. This included an altered tonotopic organization of the A1 field and reduced receptive field selectivity of individual neurons in A1. However, ABR thresholds were reported to be unaffected in these rats.

Laboratory animal studies of the effects of MeHg exposure on auditory function also report hearing loss. Rice and Gilbert (1992) examined monkeys exposed to MeHg from birth to 7 years of age and observed elevations in pure tone detection thresholds in the mid- to high-frequency range. An extension of this research examined monkeys exposed to MeHg throughout gestation and continuing postnatally until 4 years of age (Rice 1998). Pure tone detection thresholds were elevated in MeHg-exposed monkeys, and the deficits extended over an even wider range of frequencies. Two studies using a mouse model have reported elevated ABR thresholds after chronic, low-dose exposure to MeHg (Chuu et al. 2001; Huang et al. 2008).

In the present study, we extended our previous research on PCBs alone to investigate coexposure to PCBs and MeHg. Scientific evidence that PCB and MeHg exposure may have interactive effects is building. The effects of coexposure were investigated in the Oswego Newborn and Infant Development Project, a prospective birth cohort study that assessed the relationship between prenatal contaminant exposure and neuropsychological function during childhood. Although the study did not definitively identify additive or synergistic neurotoxic effects, a statistical interaction between PCB and MeHg exposure was observed, with an association between MeHg exposure and neurobehavioral end points that was stronger in children who had higher PCB exposure (Stewart et al. 2003).

Further evidence for interactive effects of PCBs and MeHg has been gained from *in vitro* studies. Bemis and Seegal (1999) reported that rat brain (striatum) tissues exposed to PCBs alone show a concentration-dependent reduction in dopamine content. Exposure to MeHg alone had no significant effect on dopamine (with the exception of the highest concentration tested). However, exposure to mixtures of PCBs and MeHg resulted in greater reductions in dopamine levels than did exposure to PCBs alone, suggesting an interaction between the two compounds. Continuing this line of research, Bemis and Seegal (2000) reported an interactive effect of PCBs and MeHg on intracellular calcium concentrations in rat cerebellar granule cells. Exposure to either PCBs or MeHg resulted in concentration-dependent calcium elevations. Low-combination doses also elevated calcium concentrations, but there was a statistical interaction identifying a difference between the effects of combined exposure and exposure to either PCBs or MeHg individually, with combined exposure resulting in greater elevations. Furthermore, high-combination doses or longer exposure durations to low-combination doses reduced calcium concentrations compared with the elevations observed after exposure to MeHg only. These studies provide evidence that PCBs and MeHg may have interactive effects that depend on the concentration and timing of the exposure.

The effects of combined exposure to PCBs and MeHg have also been studied *in vivo* in rodents. Roegge et al. (2004) investigated motor function in rats after developmental exposure to PCBs alone, MeHg alone, or PCBs and MeHg. They tested rats on cerebellar tasks, including traversing a rotating rod. Rats exposed to PCBs alone were slightly impaired on the rotating rod task, whereas rats exposed to MeHg alone did not differ from controls. However, combined exposure to PCBs and MeHg resulted in a substantial impairment relative to controls, suggesting an additive or interactive effect of the two contaminants. Littermates of those rats were examined on a working memory task using operant testing chambers (Widholm et al. 2004). PCB-only and MeHg-only groups showed performance deficits. The combined exposure group showed similar deficits, but the magnitude of the effect was no different from the effects of individual contaminant exposure. Using a mouse model, Fischer et al. (2008) report altered spontaneous behavior after exposure to PCB-153, and coexposure to MeHg exacerbated the behavioral deficit.

In the present study, we used the rat as a model to study combined exposure to PCBs and MeHg. We formulated a unique PCB mixture to match the PCB congener profile in fish from the Fox River in northeast Wisconsin (Kostyniak et al. 2005). MeHg doses were selected to achieve a ratio of PCBs to MeHg similar to that found in the fish. Thus, the study modeled exposures in a parallel study of consumers of sport-caught fish in northeast Wisconsin. The goal of this study was to determine if developmental exposure to these contaminants results in long-term effects on auditory function.

## Materials and Methods

### Animals

Primiparous female Long-Evans rats, approximately 60 days of age, were purchased in three cohorts spaced approximately 6 months apart, from Harlan (Madison, WI). We maintained animals used in these studies in facilities accredited by the Association for the Assessment and Accreditation of Laboratory Animal Care. Specifically, rats were individually housed in standard plastic shoebox cages with corn-cob bedding, in a temperature- and humidity-controlled room (22°C, 40–55% humidity) on a 12/12-hr reverse light/dark cycle (lights off at 0830 hr). Food and water were available ad libitum. Rats were fed Harlan Teklad rodent diet (W) 8604. All procedures were approved by the Institutional Animal Care and Use Committee at the University of Illinois at Urbana-Champaign and were in accordance with the guidelines of the National Institutes of Health (2002) and National Research Council (2003). We treated the rats humanely and with regard for alleviation of suffering.

### Exposure

After 2 weeks of adaptation to the vivarium, the rats in each cohort were assigned to exposure groups (distributed evenly by body weight) and given one of seven treatments consisting of PCBs and/or MeHg, or control treatment (see Table 1). Exposure began 28 days before breeding and continued until pups were weaned on postnatal day (PND) 21. We formulated the PCB mixture to mimic the congener profile found in wall-eye, a popular sport-caught fish, taken from the Fox River in northeast Wisconsin. The mixture consisted of 35% Aroclor 1242 (Monsanto lot KB 05-415; St. Louis, MO) 35% Aroclor 1248 (AccuStandards lot F-110; New Haven, CT), 15% Aroclor 1254 (Monsanto lot KB 05-612), and 15% Aroclor 1260 (AccuStandards lot 021-020). We found the mixture had relatively low aryl hydrocarbon receptor activity but high ryanodine receptor (RyR) activity (see Kostyniak et al. 2005). The doses of the PCB mixture (1 and 3 mg/kg) were selected based on the results of earlier studies assessing the *in vivo* developmental toxicity and auditory toxicity of the mixture in rats (see Kostyniak et al. 2005; Powers et al. 2006). The PCB mixture was diluted in corn oil (Mazola) and pipetted onto one-half of a vanilla wafer cookie (Keebler Golden Vanilla Wafers) at a volume of 0.4 mL/kg. To arrive at a dose of 0.4 mL/kg, the dosing solutions were mixed at concentrations of 2.5 mg/mL and 7.5 mg/mL for the PCB doses of 1 mg/kg and 3 mg/kg, respectively. The PCB-dosed cookies were fed to the female rats daily at approximately 1100 hr. Doses were adjusted daily to account for weight gain. Corn oil vehicle alone was pipetted onto cookies for rats in treatment groups that did not receive PCBs. Methylmercury was administered in the drinking water at concentrations of 1.5 and 4.5 ppm when rats were in their home cages. Adulterated water dosed with MeHg was put into plastic water bottles fitted with screw tops and ball tips to prevent spillage. Unadulterated drinking water was given to rats in treatment groups that did not receive MeHg and to all male rats used for breeding. We selected these doses of MeHg to yield a ratio of PCBs to MeHg similar to that measured in walleye from the Fox River, which was approximately 6:1 (Wisconsin Department of Natural Resources, unpublished data). The average daily amount of MeHg consumed across the entire dosing period was measured for dams in the first cohort only, which resulted in a ratio of PCBs to MeHg that was approximately 4.4:1 and 4.9:1 in the low and high combined exposure groups, respectively (see Table 1).

### Breeding, pregnancy, and weaning

Four weeks after the initiation of exposure, we paired each female with an unexposed male Long-Evans rat (Harlan, Madison, WI) in a hanging wire cage for 8 consecutive days. The breeding cages contained standard rat chow and standard tap water (ad libitum) to ensure that the males did not receive any MeHg exposure. The females were returned to their home cages each day for PCB dosing, where access to MeHg water was also available to MeHg exposure groups. We confirmed consumption of the cookie before we returned the females to the breeding cages. The females were monitored twice daily for the presence of a sperm plug in order to establish gestational day 0. Females that did not give birth were retained and their uteri examined for implantation sites.

On the day of parturition (PND 0), we examined the pups for gross abnormalities, sexed and weighed them, and noted the number of stillbirths. On PND 2, the litters were culled to 10 pups (five males and five females when possible), and small litters were cross-fostered with extra pups from the same treatment group when possible to bring them to 8–10 pups. Cross-fostered pups were marked by ear clip and were not used for auditory testing. Thereafter, pups were weighed weekly until approximately PND 70.

Because of concern about the pups being able to reach the water bottle, MeHg dosing of the dams stopped on PND 16. PCB dosing continued until the pups were weaned on PND 21. On PND 21, we euthanized the dam from each litter and recorded the liver weight and number of uterine implantation sites. Four pups per litter were retained, one male and one female for auditory testing for the present results, and one male and one female for cognitive testing (data reported elsewhere), and euthanized the remaining pups. Of these remaining pups, we obtained organ weights (brain, liver, and thymus) from one male and female per litter. We housed pups retained on the day of weaning in same-exposure, same-sex pairs or triplets with food and water ad libitum. Auditory testing began at approximately PND 200 to determine if developmental exposures resulted in permanent hearing deficits.

### Distortion product otoacoustic emissions

DPOAEs are acoustic responses generated when the cochlea is stimulated by two pure tones (called f1 and f2 primaries). The DPOAEs at the frequency 2f1 – f2 are commonly measured experimentally to assess outer hair cell function. Loss of outer hair cells or impairment of outer hair cell function results in the attenuation of DPOAE amplitudes. We conducted DPOAE testing in a sound-attenuated chamber, lined with sound-absorbing foam, within an isolated laboratory. Before testing, rats were sedated with 0.5 mL/kg ketamine/xylazine (87:13) intraperitoneally. Once sedated, rats were placed on a thermo-regulating heating pad (no. 50-7053-R, Harvard Apparatus, Holliston, MA**)** to maintain body temperature. Rats were placed on their sides and the probe was positioned in the left ear canal.

We recorded the DPOAEs using Tucker Davis Technologies (TDT; Alachua, FL) System 2/System 3 digital signal processing hardware and software. Stimuli were directed into the ear canal through a single ear probe. The probe contained two earphones and a microphone and had a soft rubber tip that sealed the ear canal from external noise. We created all DPOAE stimuli using TDT SigGen software and conducted recordings using TDT BioSig software. Details regarding the instrumentation can be found in Powers et al. (2006).

The DPOAEs were generated by simultaneously presenting two sinusoids, f1 and f2 (f2/f1 = 1.2), into the sealed ear canal of the rat. We calibrated the sound levels for the f1 and f2 primaries to 60 dB sound pressure level (SPL) and 50 dB SPL (0 dB = 20 μPa), respectively, using a pressure field microphone (no. 4192, Bruel and Kjaer, Norcross, GA) in a 2-cc calibration coupler (no. 4946, Bruel and Kjaer). The amplitude of the distortion product at the frequency defined by 2f1 – f2 was then measured by recording the pressure in the sealed ear canal. In mammals, the 2f1 – f2 distortion product is the most robust and is commonly measured as a reliable indicator of outer hair cell function (Lonsbury-Martin and Martin 1990). We selected six stimulus pairs for DPOAE testing, which included f2 = 2, 3, 4, 6, 8, and 12 kHz.

DPOAE testing consisted of measuring suprathreshold DPOAE amplitudes followed by DPOAE thresholds at each of the six stimulus pairs, beginning with 2 kHz. Each distortion product was the average response of 100 stimulus pair presentations (presentation rate = 6/sec). We repeated this so that the final distortion product represented an average of the two trials (each having 100 separate stimulus presentations). DPOAE amplitudes were calculated by subtracting surrounding noise from the 2f1 – f2 distortion product. The noise was defined as the average of the 10 neighboring frequencies (five above and five below the 2f1 – f2 distortion product). After the suprathreshold amplitudes were measured, DPOAE thresholds were then determined by reducing the f1 and f2 primaries in 5-dB steps. We defined thresholds as the lowest f2 dB level at which the 2f1 – f2 distortion product was > 6 dB above the surrounding noise.

### Auditory brainstem responses

The ABR is a far-field electrical recording of neural activity associated with the processing of acoustic information. We conducted testing in the same chamber and prepared rats for the procedure as they were for DPOAE testing. ABRs were recorded using the same TDT hardware. Stimuli were directed through a 10-cm tube, fitted with a rubber tip that allowed for the tones to be presented into the sealed ear canal. We recorded the ABR responses using needle electrodes (FE-2, Grass Technologies, West Warwick, RI**)** connected to a headstage (TDT no. HS-4) and a bioamp controller (TDT no. DB-4) that amplified the analog voltage recordings 60,000 times and band-pass filtered them with 3-dB cutoffs at 100 Hz and 3 kHz before being digitally converted (TDT no. AD-1). The sampling rates to generate stimuli and digitize the responses were 125 kHz and 20 kHz, respectively.

**Figure 1:**
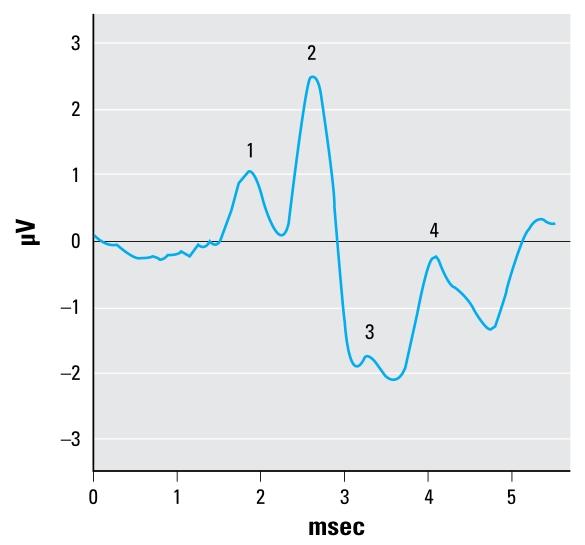
An ABR waveform generated from a control rat in response to a 6-kHz tone at 65 dB, indicating peaks 1–4.

The ABR stimulus was a 65-dB SPL sinusoid presented at a rate of 21.8/sec, with 1/3 rise, 1/3 plateau, and 1/3 fall times. The frequencies selected for testing included 1.5, 3, 6, 12, and 24 kHz, and we presented them sequentially beginning with the lowest frequency. The stimulus energy was equated for the two lowest frequencies by setting the duration to 3 msec for the 1.5 kHz stimuli and 1.5 msec for the 3 kHz stimuli (i.e., 4.5 cycles per stimulus presentation), but technical limitations prevented matching with the higher frequency stimuli. The ABR waveforms were produced via differential voltage recordings from needle electrodes placed under the skin on the scalp at the vertex (noninverting electrode) and ipsilateral mastoid (inverting electrode). The ground electrode was placed on the back of the neck. Each ABR waveform represented the average response to 500 stimulus presentations, and we determined the final analyzable ABR waveform by averaging the waveforms from two consecutive ABR trials (each the product of 500 stimulus presentations). Figure 1 shows a representative ABR waveform, indicating peaks 1–4. We measured latency and amplitude (peak to following trough) of each of the first four positive peaks of the response to the suprathreshold stimuli. ABR thresholds were then determined by reducing the stimulus level in 5-dB steps and were defined as the lowest decibel level at which peak 2 could be positively identified.

### Statistical analysis

We conducted all statistical analyses using SPSS for MS Windows (version 15.0; SPSS Inc., Chicago, IL) with statistical significance set at *p* < 0.05. DPOAE amplitudes, DPOAE thresholds, ABR thresholds, and ABR peak amplitudes and peak latencies were analyzed via separate three-way analyses of variance with exposure as a between-subjects variable, frequency as a repeated measure, litter as the unit of variance, and sex nested within litter. ABR waveforms generated by the 1.5-kHz stimuli were irregular in appearance, in that peak 2 was identifiable whereas peaks 1, 3, and 4 were not. Therefore, we used the 1.5-kHz data in the analysis of ABR thresholds, but not ABR amplitudes and latencies. We conducted post hoc comparisons, using Tukey’s test, to examine the nature of significant treatment effects obtained from the overall analyses.

## Results

### Reproductive and developmental outcomes

We saw no overt signs of clinical toxicity in the dams from any treatment group. There were no significant treatment effects related to litter size, percent males, percent live births per litter, or implantation sites. We observed no effect of treatment on timing of eye opening in pups. At PND 21, ratios of liver to body weight were significantly increased in groups exposed to PCBs, regardless of whether the dams were also treated with MeHg. This reflects liver enzyme induction in PCB-exposed pups. Ratios of brain to body weight were not affected by treatment. Ratios of thymus to body weight were significantly decreased in female pups in the 3 mg/kg PCB group, the low PCB + MeHg group, and the high PCB + MeHg group. We observed no effect of treatment on birth weight or postnatal weight gain in pups, with the exception of a slight increase at weaning (PND 21) in the 1.5 ppm MeHg group and the 1 mg/kg PCB + 1.5 ppm MeHg group. Further details pertaining to the reproductive and developmental outcomes are reported in Sable et al. (2009).

### DPOAEs

**Figure 2:**
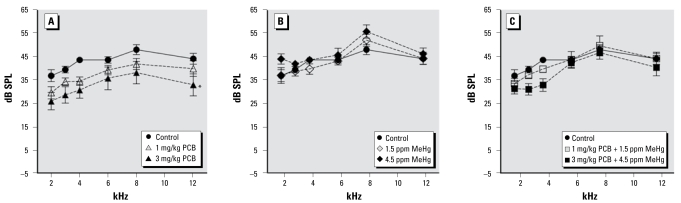
Group mean DPOAE amplitudes of control rats compared with exposed rats. Exposure to PCBs only (*A*), MeHg only (*B*), or PCBs + MeHg (*C*). *Main effect across all frequencies is significantly different from controls (*p* < 0.05).

Figure 2 illustrates DPOAE amplitudes at 2, 3, 4, 6, 8, and 12 kHz for control and PCB- and/or MeHg-exposed rats. The DPOAE amplitudes of rats exposed only to PCBs were attenuated at all frequencies. This was reflected in a significant main effect of exposure [*F* (6, 48) = 4.216; *p* = 0.002]. We also found a significant effect of frequency [(5, 240) = 50.696; *p* < 0.001] and a sex × frequency interaction [*F* (5, 240) = 4.349; *p* = 0.001]. A comparison of the sexes revealed that DPOAE amplitudes of male rats were greater at 2 kHz and lower at 12 kHz (data not shown). There was not a significant effect of sex or any significant exposure interactions. Tukey’s test revealed that DPOAE amplitudes of the 3 mg/kg PCB group were significantly lower than those of the control group (*p* = 0.016), the 1.5 ppm MeHg group (*p* = 0.029), and the 4.5 ppm MeHg group (*p* = 0.002). None of the MeHg groups or PCB + MeHg groups differed from controls.

**Figure 3:**
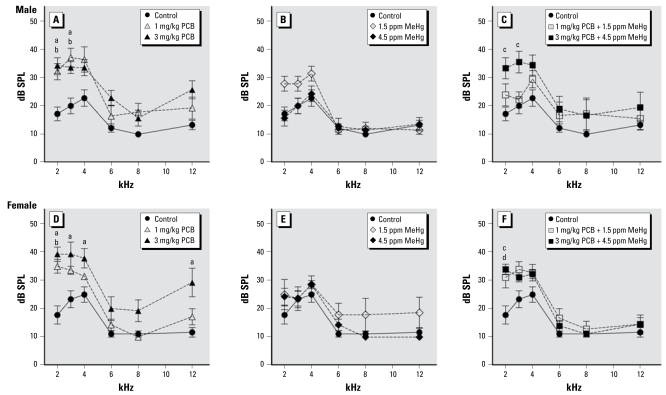
Group mean DPOAE thresholds of control rats compared with exposed rats. Exposure to PCBs only (*A*, *D*), MeHg only (*B*, *E*), or PCBs + MeHg (*C*, *F*) for male rats and female rats. Letters indicate a significant difference (*p* < 0.05) between controls and 3 mg/kg PCB (a), 1 mg/kg PCB (b), 3 mg/kg PCB + 4.5 ppm MeHg (c), and 1 mg/kg PCB + 1.5 ppm MeHg (d).

Figure 3 illustrates DPOAE thresholds at 2, 3, 4, 6, 8, and 12 kHz for control and PCB- and/or MeHg-exposed rats. DPOAE thresholds for PCB-only and PCB + MeHg rats were elevated compared with control rats. This was reflected in a significant effect of exposure [*F* (6, 48) = 6.615; *p* < 0.001]. We also found a significant effect of frequency [*F* (5, 240) = 142.352; *p* < 0.001], as well as a frequency × exposure interaction [*F* (30, 240) = 2.114; *p* = 0.001] and a frequency × sex × exposure interaction [*F* (30, 240) = 60.046; *p* = 0.012] on DPOAE thresholds. No significant effects of sex or of sex × exposure or of sex × frequency interactions were observed on DPOAE thresholds. In male rats, DPOAE thresholds at 2 kHz were elevated compared with controls in the 1 mg/kg PCB group (*p* = 0.025), the 3 mg/kg PCB group (*p* = 0.007), and the 3 mg/kg PCB + 4.5 ppm MeHg group (*p* =0.006); thresholds at 3 kHz were also elevated compared with controls in the 1 mg/kg PCB group (*p* = 0.003), the 3 mg/kg PCB group (*p* = 0.037), and the 3 mg/kg PCB + 4.5 ppm MeHg group (*p* = 0.005). We found no significant differences at 4, 6, 8, or 12 kHz in male rats. In female rats, DPOAE thresholds at 2 kHz were elevated compared with controls in the 1 mg/kg PCB group (*p* = 0.009), the 3 mg/kg PCB group (*p* = 0.001), and the 3 mg/kg PCB + 4.5 ppm MeHg group (*p* = 0.009); thresholds at 3 kHz were elevated compared with controls only in the 3 mg/kg PCB group (*p* = 0.006), and the 3 mg/kg PCB group also had elevated thresholds compared with controls at 4 kHz (*p*= 0.010) and 12 kHz (*p*= 0.008).

### ABRs

**Figure 4:**
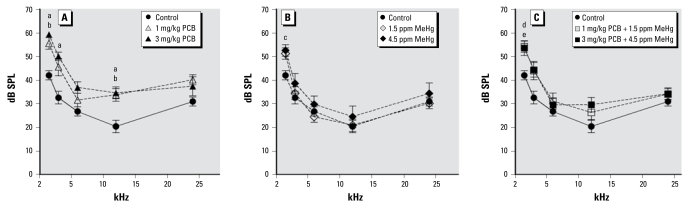
Group mean ABR thresholds of control rats compared with exposed rats. Exposure to PCBs only (*A*), MeHg only (*B*), or PCBs + MeHg (*C*). Letters indicate a significant difference (*p* < 0.05) between controls and 3 mg/kg PCB (a), 1 mg/kg PCB (b), 4.5 ppm MeHg (c), 1 mg/kg PCB + 1.5 ppm MeHg (d), and 3 mg/kg PCB + 4.5 ppm MeHg (e).

Figure 4 illustrates ABR thresholds at 1.5, 3, 6, 12, and 24 kHz for control and PCB- and/or MeHg-exposed rats. ABR thresholds for PCB-only and PCB + MeHg rats were elevated compared with control rats. This was reflected in a significant effect of exposure [*F* (6, 48) = 3.209; *p* < 0.010]. We also found a significant effect of frequency [*F* (4, 192) = 333.027; *p* < 0.001] and a significant effect of sex [*F* (1, 48) = 8.983; *p* = 0.004], as well as a frequency × exposure interaction [*F* (24, 192) = 2.375; *p* = 0.001]. The effect of sex resulted from the females having lower ABR thresholds compared with the males. There were no observed sex × exposure or sex × frequency interactions. At 1.5 kHz, ABR thresholds were significantly elevated compared with control rats in the 1 mg/kg PCB group (*p* = 0.004), the 3 mg/kg PCB group (*p* < 0.001), the 4.5 ppm MeHg group (*p* = 0.050), the 1 mg/kg PCB + 1.5 ppm MeHg group (*p* = 0.007), and the 3 mg/kg PCB + 4.5 ppm MeHg group (*p* = 0.012). At 3 kHz, ABR thresholds were significantly elevated compared with control rats only in the 3 mg/kg PCB group (*p* = 0.007). At 6 kHz, we found no significant differences in ABR thresholds. At 12 kHz, ABR thresholds were significantly elevated compared with control rats in the 1 mg/kg PCB group (*p* = 0.048) and the 3 mg/kg PCB group (*p* = 0.028). At 24 kHz, there were no significant differences in ABR thresholds.

**Figure 5:**
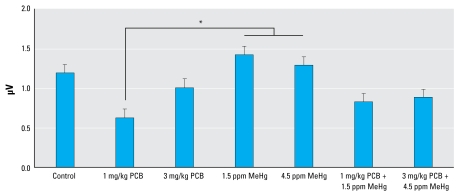
Group mean ABR peak 1 amplitude across all frequencies. *Significant difference (*p* < 0.05).

Figure 5 illustrates ABR peak 1 amplitude across all frequencies for each experimental group. Statistical analysis of ABR peak amplitudes revealed a difference at peak 1. Analysis of peak 1 showed a significant main effect of exposure [*F* (6, 48) = 2.826; *p* = 0.019]. We also found a significant effect of frequency [*F* (3, 144) = 97.862; *p* < 0.001] and a significant effect of sex [*F* (1, 48) = 22.005; *p* < 0.001], as well as a sex × frequency interaction [*F* (3, 144) = 4.539; *p* = 0.005]. The sex effect was due to greater ABR amplitudes in female rats. There were no significant sex × exposure or frequency × exposure inter actions on ABR peak 1 amplitudes. Tukey’s test revealed that ABR peak 1 amplitudes from the 1 mg/kg PCB group were significantly decreased compared with the 1.5 ppm MeHg group (*p* = 0.039) and the 4.5 ppm MeHg group (*p* = 0.036), but none of the groups differed significantly from the controls. We found no significant effects related to exposure in the analysis of the amplitudes of ABR peaks 2, 3, or 4. Statistical analysis of ABR peak latencies revealed no significant effect of exposure at any of the four measured peaks.

## Discussion

The present study confirmed and extended the results obtained from previous studies conducted in our laboratory (Lasky et al. 2002; Powers et al. 2006). Developmental PCB exposure resulted in permanent auditory deficits in rats. PCB exposure was associated with attenuation of DPOAE amplitudes and elevation of DPOAE thresholds. PCB exposure was also associated with elevation of ABR thresholds. In contrast, developmental exposure to MeHg did not induce permanent auditory deficits. Unexpectedly, we found an apparent interaction such that the effects of PCB exposure were less pronounced in rats exposed to PCBs in combination with MeHg.

Rats maternally exposed to the Fox River PCB mixture showed reduced DPOAE amplitudes, reflecting loss or impaired function of outer hair cells in the organ of Corti. As was observed previously (Powers et al. 2006), both the 1 and 3 mg/kg PCB dose groups appeared to have attenuated DPOAEs compared with controls, but the difference reached statistical significance only in the 3 mg/kg group. DPOAE thresholds were elevated, but unlike results from the earlier study, both dose groups showed significant differences from controls. DPOAE amplitudes and thresholds were unaffected by either of the MeHg doses.

Intriguingly, the effects of PCB exposure on DPOAEs appeared to be attenuated by coexposure to MeHg. DPOAE amplitudes from both combined exposure groups were similar to those of controls, although the high combined exposure group appeared to have slightly attenuated DPOAEs at 2, 3, and 4 kHz. DPOAE thresholds were elevated in male rats from the 3 mg/kg PCB + 4.5 ppm MeHg group at 2 and 3 kHz; in females, both combined exposure groups had elevated thresholds at 2 kHz but showed no significant effects at other frequencies. Low-frequency hearing may be particularly sensitive to PCB exposure (Crofton and Rice 1999; Herr et al. 1996), and this may explain why low-frequency deficits were not attenuated in the combined exposure groups. In general, the DPOAE threshold elevations in the combined exposure groups appeared similar to, but less pronounced than, the effects observed in rats exposed to PCBs alone.

Measurement of ABR thresholds yielded similar results. PCB-only groups had elevated ABR thresholds at 1.5, 3, and 12 kHz. The results were in slight contrast to our previous study, in which the 1 mg/kg PCB group did not differ significantly from controls. The 4.5 ppm MeHg group had elevated ABR thresholds at 1.5 kHz, but no differences at any other frequency. Again, the effects of PCB exposure appeared to be attenuated by co-exposure to MeHg. Both combined exposure groups had elevated ABR thresholds at 1.5 kHz but did not differ from controls at other frequencies. Again, this may reflect particular sensitivity of low-frequency hearing to PCB exposure.

ABR peak amplitudes were relatively unaffected by the exposure conditions. Although the amplitude of peak 1 was significantly lower in the 1 mg/kg PCB group compared with the two MeHg-only groups, none of the exposure groups differed significantly from controls. We are unsure of the significance of this finding because the data showed both a high degree of variability and large sex differences. The ABR peak latencies were unaffected by any of the exposure conditions.

Thresholds for DPOAEs and ABRs were elevated at some frequencies in the 1 mg/kg PCB group. Although the threshold elevations in this group were limited primarily to low frequencies, the effects contrast our previous study in which the 1 mg/kg PCB group did not differ from controls (Powers et al. 2006). In the time between the two studies, our laboratory was relocated to a new testing room that was more isolated from outside noise, and we found that the DPOAE and ABR thresholds of control rats were lower than those of controls in the previous study. Subtle threshold elevations we observed in the 1 mg/kg PCB group may have been masked by greater background noise in the previous study.

The PCB-induced auditory deficits were consistent with earlier animal models of PCB exposure. Previous studies have reported elevated low-frequency (1 kHz) hearing thresholds for the acoustic startle response in rats developmentally exposed to Aroclor 1254 (Goldey et al. 1995; Goldey and Crofton 1998) or PCB-126 (Crofton and Rice 1999). The hearing deficit was later associated with a loss of outer hair cells in the upper middle and apical region of the organ of Corti (Crofton et al. 2000). Our laboratory helped confirm a cochlear site of action by demonstrating DPOAE deficits at low frequencies in rats developmentally exposed to Aroclor 1254 (Lasky et al. 2002). Developmental exposure to the Fox River PCB mixture caused similar deficits that extended across a broader range of frequencies (Powers et al. 2006). The observation that PCB exposure resulted in ABR threshold elevations is also consistent with an effect at the level of the cochlea. However, a recent study of rats with early exposure to PCB-95 showed an alteration in the tonotopic mapping of the primary auditory cortex, which occurred in rats that did not appear to have peripheral hearing deficits (Kenet et al. 2007).

The various PCB mixtures and individual congeners may be exerting their effects through different mechanisms. PCB-induced reduction of circulating thyroid hormone levels may have a significant role in hearing loss. Hypothyroidism in pregnant women has long been known to cause hearing deficits in children (Sher et al. 1998). Animal models have also shown that maternal hypothyroidism causes structural and functional deficits in the auditory system (Uziel et al. 1980, 1981), including impaired DPOAEs (Knipper et al. 2000). Furthermore, thyroxine replacement in the offspring of rats exposed to Aroclor 1254 partially ameliorated hypothyroxinemia and hearing loss (Goldey and Crofton 1998).

However, PCBs may also induce auditory deficits via their effects on RyR activity. PCBs can act on the RyR by stabilizing a high-affinity binding conformation, which results in increased intracellular calcium concentrations (Wong et al. 1997). Alterations of intra-cellular calcium concentrations could have detrimental effects on cells in the central auditory pathway. Although speculative, both thyroid-hormone–mediated and RyR-mediated mechanisms may be involved in PCB-induced hearing loss, and individual PCB congeners may differ in the extent of involvement. For example, PCB-95 is a potent activator of the RyR but has only modest effects on thyroid hormone concentrations. This may explain the observations by Kenet et al. (2007). The altered tonotopic mapping of the auditory cortex may have resulted from disruption of RyR signaling in cortical neurons, whereas cochlear function remained normal because of a lack of effect on thyroid hormone.

We did not observe hearing deficits in rats developmentally exposed to MeHg, with the exception of elevated ABR thresholds at 1.5 kHz. Previous rodent models have reported MeHg-induced hearing deficits, but the studies did not involve developmental exposure, and deficits were not permanent (Chuu et al. 2001; Huang et al 2008). Primate models have also shown MeHg-induced hearing impairment, but this followed lengthy exposure durations that continued throughout life (Rice 1998; Rice and Gilbert 1992). MeHg may induce only transient hearing deficits from which there can be functional recovery.

A surprising result of the present study was that coexposure to MeHg appeared to attenuate PCB-induced hearing deficits. A similar attenuation of PCB-induced effects was observed when littermates of the animals tested in the present study were tested for cognitive function (Sable at al. 2009). Rats were trained on a differential reinforcement of low rates operant task. PCB-exposed groups had a lower ratio of reinforced to non-reinforced responses than controls. MeHg-exposed rats were not impaired on this task. However, groups exposed to combined doses of PCBs and MeHg did not show the deficits observed in rats exposed to PCBs alone. Previous studies in our laboratory showed that coexposure to PCBs and MeHg exacerbated PCB-induced motor impairments on a rotating rod task (Roegge et al. 2004) but did not exacerbate PCB-induced impairments on a spatial alternation task (Widholm et al. 2004). However, those studies used a different PCB mixture and a different dose of MeHg. Interactive effects of coexposure to PCBs and MeHg have also been observed *in vitro* (Bemis and Seegal 2000). In rat brain tissue, exposure to either PCBs or MeHg caused an elevation of intracellular calcium concentrations. A statistical interaction revealed a difference between the effects of coexposure and the effects of exposure to either of the two chemicals individually, with co exposure having greater effects. Additionally, high-combination doses or longer exposure durations to low-combination doses reduced calcium concentrations compared with elevations observed after exposure to MeHg only. It is evident that PCBs and MeHg can interact in complex ways to cause functional changes, but the nature of this interaction remains unresolved.

Although the doses used in this study were significantly higher than would typically be encountered in children from environmental exposure, basic principles of allometric scaling dictate that rodents must receive much higher doses than humans in order to achieve comparable body burdens. Although it is difficult to make cross-species comparisons of PCB dose, we estimate that body burdens in the present study are similar to body burdens that have been documented in more highly PCB-exposed human populations in other parts of the world, such as populations living near former PCB manufacturing sites in eastern Europe (Cerna et al. 2008).

Both PCB and MeHg exposure have been associated with hearing deficits in human epidemiologic studies, but the data are very limited because auditory function has not been a primary focus of these studies (Wigle et al. 2008). Higher exposures have been associated with elevated hearing thresholds, using traditional audiometric methods, but the effects were observed only at specific frequencies and only unilaterally. Our studies have indicated that DPOAEs and ABRs are sensitive measures of PCB-induced auditory impairments. It would be advisable that future epidemiologic studies use these methods because they can easily measure auditory function in human adults and children and they may be particularly sensitive to PCB-induced effects.

## Figures and Tables

**Table 1:** Number of litters per treatment group and MeHg intake.

Treatment group	No.	MeHg intake (μg/kg)^a^
Control	9	N/A
1 mg/kg PCB	7	N/A
3 mg/kg PCB	7	N/A
1.5 ppm MeHg	7	209.6 ± 18.4
4.5 ppm MeHg	7	715.7 ± 58.2
1 mg/kg PCB + 1.5 ppm MeHg	9	228.8 ± 22.0
3 mg/kg PCB + 4.5 ppm MeHg	9	611.9 ± 55.1

a MeHg dissolved in drinking water reported as average daily intake (mean ± SE) for the entire dosing period for cohort 1.
